# Arctic Oil Drilling Plans Raise Environmental Health Concerns

**DOI:** 10.1289/ehp.119-a116

**Published:** 2011-03

**Authors:** Charles W. Schmidt

**Affiliations:** **Charles W. Schmidt**, MS, An award-winning science writer from Portland, ME, has written for *Discover Magazine, Science*, and *Nature Medicine*

As Royal Dutch Shell and other oil companies prepare to drill offshore in the Alaskan Outer Continental Shelf (OCS), a new report commissioned by the Washington, DC–based Pew Environment Group concludes current response capabilities aren’t adequate to contain and clean up a major spill in the area.^1^ Marilyn Heiman, who directs the group’s U.S. Arctic program, says drilling on the Alaskan OCS requires a science-based precautionary approach. “And right now, we don’t know enough about the potential consequences of a spill to the ecosystem,” she says.

Chuck Clusen, director of the national parks and Alaska projects at the Natural Resources Defense Council, says that by aggregating the technical concerns associated with offshore Arctic oil and gas activities, the report provides a much-needed resource for officials and the public. “The issues it raises need to be addressed before any further oil and gas activities go forward,” he says.

The report states emphatically that spill prevention and response must fit Arctic-specific risks. Where extreme depths are the primary challenge for offshore drilling in the Gulf of Mexico, drilling and spill cleanup in the OCS will be challenged by ice cover, subzero temperatures, and harsh weather. Many environmentalists are concerned industrial drilling operations could threaten or harass the region’s wildlife, including bowhead whales. Moreover, Alaska Native populations that rely in part on marine mammals for subsistence could be affected if those mammals move farther offshore to avoid boat traffic. Spilled oil, meanwhile, persists much longer in Arctic waters than in warmer seas; microbes are slow to degrade oil under cold conditions, and the oil’s most toxic fractions— namely, benzene, toluene, ethylbenzene, and xylene—can persist for long durations before evaporating, posing risks to aquatic species, according to Ronald Atlas, a professor of biology at the University of Louisville. Shell plans to drill during the “open-water” season, which lasts approximately from June through late October. After that, the OCS begins to freeze over.

According to experts cited in the Pew report, surface ice interferes with the booms, skimmers, and other tools used in mechanical oil recovery. Oil trapped under pack ice in the winter can’t be accessed for *in situ* burning, the report states. What’s more, Heiman says, “There’s no proven response method for cleaning [up] an oil spill in the midst of broken ice. Shell says they’ve done studies that show you can do it, but those are under highly controlled experimental conditions. We have no idea what would happen in a real-world scenario.”

Officials with the Alaska region of the Bureau of Ocean Energy Management, Regulation, and Enforcement see things differently. They would not comment on the Pew report directly. But an agency official did describe Shell’s plan—which includes an on-site oil spill response fleet, near-shore barges, and responders drawn partially from local oil cleanup companies,^2^ including Alaska Clean Seas of Anchorage—as robust and well-rounded.

“They’re prepared to use ice breakers to open up areas for skimming, and they have other methods for cleaning oil when the ice goes solid,” the official says. “For instance, they can drill through the ice, allow oil to rise to the surface for cleaning, and they can burn what’s trapped in the ice later when the ice begins to melt.” The oil clings to the lower ice surface that touches the water and travels with the ice. When the ice melts, the oil is released.

The report does not address potential health risks from offshore oil development to indigenous populations, including the Inupiat Community of the Arctic Slope, an Alaska Native tribe. Jonathan Jemming, an environmental attorney in Salt Lake City, Utah, and former offshore counsel with the North Slope Borough Law Department in Barrow, Alaska, says these groups already have disproportionately high rates of cardiopulmonary ailments, an Arctic reality he says has never been fully analyzed.

“We are also seeing higher concentrations of nitrogen dioxide and particulate matter on village air monitors, but we’re not entirely sure where it’s coming from,” Jemming says. “What native communities need is for the industry and the federal government to do comprehensive air modeling to determine who the contributors are and how much more [pollution] they can add without compromising the health of the Arctic people.”

Shell’s drilling plans are now held up because of its offshore air emissions permit, which is under review by the U.S. Environmental Protection Agency (EPA). The permit had been approved by the EPA last year. But then the Inupiat Community of the Arctic Slope, the Alaskan Eskimo Whaling Commission, and several local environmental groups challenged the permit, arguing in part that it doesn’t take into account the EPA’s more stringent National Ambient Air Quality Standard for nitrogen dioxide, which was issued 9 February 2010.^3^ The EPA’s Environmental Appeals Board— an independent review board within the agency—agreed and remanded the permit back to the agency on 30 December 2010.

On 3 February 2011, Pete Slaiby, vice president of Shell Alaska, announced the company’s plan to drill during the summer of 2011 would be delayed until issues with its air permit could be resolved.^4^ Shell now hopes to begin drilling in 2012, Slaiby said during a press conference to announce the delay.

According to Jemming, ice-breaking ships—not the drill rigs themselves—are expected to contribute as much as 90% of air emissions from offshore oil and gas exploration. “These ships, especially older models, are huge beasts of the ocean,” he says. “And it takes a lot of propulsion to move the ice around.”

Stakeholders have argued about how much pollution those ships might release. Jemming claims the EPA’s own analysis predicted that one season’s exploration emissions could rival those generated by 3 million passenger automobiles a year. However, two years ago Shell committed to fuel ice-breakers with ultra-low sulfur diesel,^5^ a move Jemming says could greatly reduce the overall emission levels.

Meanwhile, on 11 January 2011 the National Commission on the BP *Deepwater Horizon* Oil Spill and Offshore Drilling, established by President Obama, released its final report.^6^ In the few of its more than 300 pages that were devoted specifically to Alaskan OCS drilling, the report reported an “optimistic” prediction by Shell that drilling in the region could ultimately peak at 1.8 million barrels per day. The commission cited numerous concerns also raised by the Pew Environment Group: hurricane-strength storms, ice, polar darkness,^7^ pervasive fog, and “serious questions about how to access spilled oil when the area is iced over or in seasonal slushy conditions.” Nevertheless, the report also stated that the need for additional research on how to manage these challenges should not pose a “*de-facto* moratorium on activity in the Arctic.”^6^

“If Shell is allowed to go forward and they strike significant amounts of oil, all the other companies are going to head up there full blast,” Clusen says. “And then the federal government is going to have to respond to a slew of requests and demands from the oil industry.”

## Figures and Tables

**Figure:**
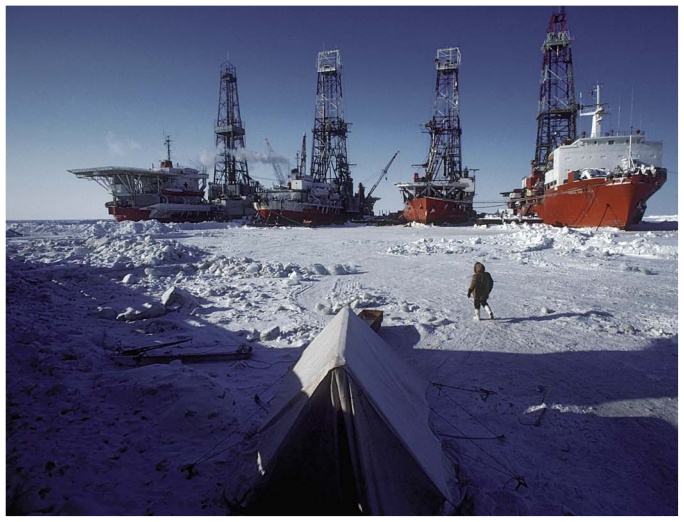
Ice-breaking ships, described as “huge beasts of the ocean,” may contribute as much as 90% of air emissions from offshore oil and gas exploration—although the choice of fuel used in the ships can make a significant difference.
